# Investigation of composition, antioxidant, antimicrobial and cytotoxic characteristics from *Juniperus sabina* and *Ferula communis* extracts

**DOI:** 10.1038/s41598-023-34281-x

**Published:** 2023-05-03

**Authors:** Doga Kavaz, Razan El Khaled El Faraj

**Affiliations:** 1 Department of Bioengineering, Cyprus International University, Northern Cyprus Via Mersin 10, Haspolat, Nicosia Turkey; 2 Biotechnology Research Centre, Cyprus International University, Northern Cyprus Via Mersin 10, Haspolat, Nicosia Turkey

**Keywords:** Antimicrobials, Plant sciences, Natural variation in plants

## Abstract

Plants have been one the most valuable sources of biologically active compounds. This study investigates the chemical composition, as well as the antioxidant, antimicrobial, and cytotoxic activities of methanolic and ethanolic extracts from *Juniperus sabina* and *Ferula communis* leaves, grown in Cyprus. Total phenolic and flavonoids content of methanol and ethanol extracts were quantified. Chemical constituents of the leaf extracts were analysed using gas chromatography/mass spectrometry (GC/MS). Mome inositol was the predominant component in the *J. Sabina*’s extracts. The most dominant component in *F. communis* ethanolic extract was phytol, while in FCL methanolic extract 1,3,4,5 tetrahydroxycyclohexanecarboxylic acid. Antioxidant activities were evaluated by 1, 1-diphenyl-2-picrylhydrazyl (DPPH) free radical-scavenging ability. Antioxidant activity results revealed concentration dependent activity for methanolic and ethanolic extracts from the plant leaves. Antibacterial activity of plant extracts was tested against Gram-negative and Gram-positive bacteria using disk diffusion and minimal inhibitory concentration methods. Cytotoxic activity of plant extracts were evaluated on MCF-7 and MDA-MB-231 breast cancer cell lines, where they demonstrated their potential on the viability of both cell lines. The biological activity revealed by plants is due to the bioactive compounds found in the extracts. These bioactive components could be used as anticancer drug candidates.

## Introduction

For public health, the WHO assessed cancer as the most common cause of death in persons younger than 70 years of age in 2015 and it is one of the significant medical issues in Turkey and across the globe. In 2012, cancer accounted for almost 8.2 million deaths worldwide^1^. A review by Turkey's Health of Ministry demonstrated 396 thousand cancer cases between 2000 and 2006, with 140 thousand deaths. Turkey has reported cancer as the second highest cause of mortalities in the last decade, following circulatory system disease^2^. Breast cancer, the most prominent malignant tumor in women, has proved difficult to treat^3^. The most widely used and effective treatments also cause discomfort, amongst other adverse side effects, in patients. Methods to decrease patient distress whilst increasing therapeutic effect has been under development^4^. Developments relating to human health and treatment of disease are increasing daily. The use of synthetic materials in industry, medicine, and agriculture also threatens human health and the environment^5^. Therefore, many diseases, are emerging and being identified as a concern. Changing synthetic materials, which cause such conditions, to natural herbal products has yet to be sufficiently accomplished to cure such illnesses. The potential for the healing effects of herbs, attributed to secondary metabolites, is vast, with many such plants still needing to be more active^6–8^.

Since early human civilization, plant-derived natural products have been used for their therapeutic properties and as a primary source of drugs. Medicinal plants are known for their antioxidant properties, which serve in the development of novel medications^9^. Increasing interest in the secondary metabolites of plants has shown their significant biological activities and the importance of their structural arrangements and variety to their therapeutic properties^10^. Hence, there is an increasing demand for the identification of effective and safe naturally occurring products. Furthermore, secondary metabolites play a crucial function in the suppression of a variety of cancers. As a result, studying the biological activities of plants is critical for verifying a herb's properties and its therapeutic use in human health.

The genus *Juniperus* (family Cupressaceae) comprises about 60 species^11^. *J. sabina* L. (JSL), also called savin or *Savin Juniper*, is found abundantly in south-west and south-east Europe, in Ukraine, Russia, Iran, and Turkey, where it is named “Ardıç Ağacı”, as well as Kazakhstan and China^12^. A broad range of critical biological activities have been detected in this plant, such as antioxidant^13^, antiviral, antibacterial^14^, anti-inflammatory, hypotensive, abortifacient, anti-nociceptive^15^ and anticancer^16^.

The genus *Ferula* (Family Apiaceae) comprises about 170 species^17^. *F. communis* L. (FCL) is a flowering plant, 1–2.5 m high, herbaceous, with smooth leaves and a large sheath. It is distributed from central Asia, throughout the Mediterranean, to North Africa and is considered endemic to Cyprus^18^. It has been historically used for many medical purposes and has been prescribed to treat a variety of diseases as an antiseptic and antihysteric and as a treatment for headaches, dysentery, dizziness, digestive disorders, rheumatism, arthritis, and toothache. In addition, previous research has proven that some *Ferula* plants have practical therapeutic usage in cancer treatment^19^.

The island of Cyprus contains a plethora of plant species whose therapeutic value has yet to be unlocked. However, there are very few reports on the potential of *J. sabina* and *F. communis* in this regard. Therefore, this study determined the composition of methanolic and ethanolic extracts from these plants’ leaves. Additionally, antioxidant and antimicrobial activity, as well as the total phenolic and flavonoids content were investigated. Antiproliferation activity was also evaluated for the first time against both strongly (MDA-MB-231) and weakly (MCF-7) metastatic breast cancer cell lines.

## Materials and methods

### Chemicals

All chemicals were purchased from Merck (Germany) and Sigma Aldrich.

### Plant sample collection

*Juniperus sabina* and *Ferula communis* leaves were collected from North Cyprus, Hisarköy (Kampyli: 35°18′05″ N 33°06′25″ E), respectively, in October 2019. The collection of plant material complies with relevant institutional, national, and international guidelines and legislation. Dean of Faculty of Agricultural Sciences and Technologies Prof. Dr. İbrahim Baktir in, Cyprus International University identified the plants. The plant material was air-dried in a well-ventilated shadow place, ground into powder, and deposited in Cyprus International University Public Herbarium (*F. communis* L. (FCL) herbarium number: CIUH-232; *J. sabina* L. (JSL) herbarium number: CIUH-345). No special permission is needed for collection purposes.

### Preparation of methanolic and ethanolic extracts of JSL and FCL

*Juniperus sabina* and *F. communis* (50 g each) were extracted separately using 250 ml of 95% methanol or 95% ethanol at a temperature below the boiling point of the solvents. Obtained mixtures were filtered using Whatman filter paper No. 1 and concentrated under reduced pressure at 45 °C using the rotary evaporator (Heidolph, Germany), leaving a viscous residue, dark green with an aromatic odor. These samples were stored in the refrigerator at 4 °C for further analysis.

### Total phenolic content

The total phenolic content was determined with some slight modifications^20^. Appropriately diluted extract (100 µl) was mixed with 200 µl of an undiluted Folin-Ciocalteu reagent for 5 min. 1 ml of 20% Na_2_CO_3_ (Sodium carbonate) was added to the mixtures and the total volume was adjusted to 10 ml with distilled water. This was incubated at room temperature in darkness for 2 h, and the absorbance measured at 765 nm using a UV Visible Spectrophotometer (UV-2450). The results were estimated as gallic acid equivalent (GAE), expressed as mg GAE/g. The calibration curve range was 1.25–20 mg/ml (R^2^ = 0.99). The data are presented as means ± standard deviation (SD) from three biological repeats.

### Total flavonoid content

Total flavonoid content was calculated by the aluminum chloride (AlCl_3_) colorimetric method with some modifications^21^. Briefly, 1 ml of aliquots, 4 ml of distilled water and 0.3 ml of 5% NaNO_2_ (sodium chloride) were placed in separate test tubes. After 5 min, 0.3 ml of 10% AlCl_3_ and 2 ml of 1 M NaOH (sodium hydroxide) were added to the mixture. The mixture was adjusted to 10 ml with distilled water and mixed well, developing an orange/yellow color. Using the spectrophotometer (UV-2450), the absorbance was measured at 510 nm wavelength versus a prepared blank. The total flavonoid contents of each JSL and FCL plant extract were expressed as milligrams of quercetin equivalents per gram of dry matter (mg QE/g) through the calibration curve with quercetin. All results are presented as means (± SD) from three biological repeats.

### GC–MS analysis

The components of methanolic and ethanolic extracts of both JSL and FCL were identified using gas chromatography-mass spectrometry (GC–MS) analysis. The GC–MS apparatus used was Shimadzu QP- 2010 equipped with a fused-silica column. The operating conditions were: flow rate of helium gas 1 ml/min; oven temperature 50–310 °C with a rate of 8 °C/min; injector temperature 275 °C; split injection mode of ratio 1:10; pressure 112.2 kPa; Ionization energy 70 eV.

The extract components were identified from the retention indices (RI) obtained by computer matching with the MSdata bank (Wiley Library), and relative percentages were calculated.

### DPPH radical scavenging assay

The total antioxidant activity of each sample was determined using the 1, 1-diphenyl-2-picrylhydrazyl (DPPH) free radical scavenging activity assay^22^. The DPPH was freshly made using pure ethanol of 0.004% w/v. A volume of 1 ml of diluted samples (concentrations 100, 50, 25, 12.5, 6.25 µg/ml) were mixed with 1 ml of DPPH. These were incubated in darkness for 30 min, at 25 °C, before the absorbance of each sample was measured at 517 nm. A blank was prepared by mixing 0.5 ml of DPPH solution with 0.5 ml of ethanol. A positive control of ascorbic acid was prepared to compare the results of decreased absorption induced by the samples. The capability to scavenge 50% of DPPH was calculated using Eq. (1). All results presented are means (± SD) from three biological repeats.

1 $$ {\text{Percentage}}\;{\text{Inhibition}}\;{\text{ratio}}\;\left( \% \right) = \left[ {\left( {{\text{A}}\;{\text{control}} - {\text{A}}\;{\text{sample}}} \right)/\left( {{\text{A}}\;{\text{control}}} \right)} \right] \times {1}00 $$

### Antimicrobial activity

#### Microorganisms

The antimicrobial activity of methanolic and ethanolic extracts obtained from both JSL and FCL were determined using four strains of microorganisms: *E. coli* O157:H7 (932), *S. typhimurium* (B-4420), *B. cereus* (ATCC 7064) and *S. aureus* (6538 P). “Minimum inhibitory concentrations (MIC)” and “Disk diffusion method” were used to determine antimicrobial activity. Nutrient agar and nutrient broth were prepared according to the manufacturer and sterilized in the autoclave for 20 min at 121 °C. The final microorganism concentration was adjusted to 0.5 McFarland Standard (1.5 × 108 CFU/ml).

#### Minimum inhibitory concentrations (MIC)

MIC of methanolic and ethanolic extracts of JSL and FCL were estimated using the broth dilution method. The diluted sections of five concentrations (6.25, 12.5, 25, 50 and 100 mg/ml) were prepared using 30% dimethyl sulfoxide (DMSO)^23^. The MIC was estimated using 3 ml of sterile nutrient broth cultured with 1 ml of the bacterial suspension. 1 ml of each extract concentration was added to the mixture and incubated for 24 h at 37 °C before the results were collected. Each assay was performed in triplicate.

#### Disc diffusion method

Nutrient agar plates were inoculated with 100 µl of prepared bacterial suspension using a sterile wire loop swabbed on the surface of the agar plates. Sterile 6 mm discs were soaked with 15 µl of one of the five concentrations of methanolic and ethanolic extracts of JSL and FCL. The diluted extracts of five different concentrations (6.25, 12.5, 25, 50 and 100 mg/ml) were prepared by using 30% DMSO^24^. On each plate, five 6 mm discs were placed on the surface of the nutrient agar. Plates were incubated for 24 h at 37 °C. DMSO was used as a negative control. The diameter of the zones of inhibition (ZI) around the discs determined bacterial growth inhibition. The zones of inhibition were measured using a ruler and recorded in millimeters. Each assay was performed in triplicate with DMSO as a negative control. The effects of each concentration of extracts tested on the bacteria were compared with sensitivity to antibiotics chloramphenicol and amoxicillin.

### Cytotoxic activities

#### Cell lines and cell culture conditions

Human breast cancer cell lines MDA-MB-231 and MCF-7 were obtained from Imperial College London, UK (courtesy of Prof. Dr. Mustafa Djamgoz). Both cancer cell lines were cultured in Dulbecco's Modified Eagle Medium (DMEM) supplemented with 4 mM l-glutamine and 10% fetal bovine serum. The cells were harvested at a sub-confluence of 80–100% using 0.05% trypsin/EDTA. The cells were incubated in 5% CO_2_ and 100% relative humidity at 37 °C.

#### In vitro cytotoxicity profile

Antiproliferative activities of methanolic extracts of JSL and FCL were determined against MDA-MB-231 and MCF-7 using Trypan blue exclusion assay^25^. The antiproliferative activities of the extracts and the controls were investigated at five concentrations (10, 20, 50, 150 µg/ml). 35 mm sterile dishes (3 dishes per each condition) were used to grow cultured cells at a density of 3 × 104 cells per dish, incubated overnight to settle. Cells were treated with extracts for 24 h in a humidified incubator of 5% CO_2_ at 37 °C. The medium was removed, and 0.4% Trypan blue solution was added. After 10 min, the diluted Trypan blue was removed. Around 15 fields of view were counted randomly using an inverted microscope (Leica, Germany) to estimate the percentage of cell viability. Control dishes contained cells with DMSO^24^. Each assay was performed at least in triplicate.

#### Statistical analysis

All experiments were performed at least in triplicate. The results obtained were expressed as the mean values ± standard deviation (SD) for all experiments. Student's t-test determined significant differences to the control value for each test. The differences were considered statistically significant from the control at p < 0.05. Bar graphs show error bars for the ± standard error of the mean.

## Results and discussion

### Total phenolic content

Table 1 shows the quantitative analysis of the total phenolic content of methanolic and ethanolic extracts from JSL and FCL. The total phenolic content for both plants was found to be higher in ethanolic extracts compared to methanolic extracts. For methanolic extracts, the phenolic content of JSL (77.02 ± 3.3 mg GAE/g) was higher than FCL (17.97 ± 0.45 mg GAE/g). For ethanolic extracts, the phenolic content of JSL (124.73 ± 1.9 mg GAE/g) was also higher than FCL (91.5 ± 3.01 mg GAE/g). The quantitative analysis of the total phenolic content of both extracts from JSL showed a higher content (methanolic 77.02 and ethanolic 124.73 mg GAE/g), than those revealed by FCL extracts (methanolic 17.97 and ethanolic 91.5 mg GAE/g). Ethanolic extracts of both plants provided a higher extraction of phytochemicals than methanolic extracts. *F. communis* from Jordan was previously shown to have a similar total phenolic content in the methanol extract (18.4 mg GAE/g)^26^. According to Öztürk et al., *J. sabina* showed lower total phenolic content of methanolic extracts from plants collected in the Karabük province in Turkey (31.58 mg GAE/g), as compared to this study^27^. Zengin et al., studied the difference in total phenolic content of *F. halophila* extracted from three solvents, with the highest phenolic content observed from the acetone extract, followed by methanol, then chloroform^28^.

**Table 1:** Quantitative determination of Total Phenolic Content (Gallic acid was used as the standard) and total Flavonoid content (Quercetin was used as a standard) in the leaves of *Juniperus sabina* and *Ferula communis* of both methanolic and ethanolic extracts.

Type of extract	Total phenolic content (mg GAE/g)		Total flavonoid content (mg QE/g)	
	JSL	FCL	JSL	FCL
Methanol extract	77.02 ± 3.3	17.97 ± 0.45	77.02 ± 3.3	17.97 ± 0.45
Ethanol extract	124.73 ± 1.9	91.5 ± 3.01	124.73 ± 1.9	91.5 ± 3.01

Values are means ± SD, (n = 3).

*JSL* Juniperus sabina leaves, *FCL* Ferula communis leaves, *GAE* gallic acid equivalent expressed as mg GAE/g, *QE* Quercetin equivalent expressed as mg QE/g.

### Total flavonoid content

Table 1 shows the quantitative analysis of total flavonoids of both methanolic and ethanolic extracts from JSL and FCL. Higher total flavonoid content was found for both JSL and FCL in ethanolic extracts than methanolic extracts. For methanolic extracts, the flavonoid content of JSL (77.02 ± 3.3 mg QE/g) was higher than FCL (17.97 ± 0.45 mg QE/g). Among the ethanolic extracts, the flavonoid content of JSL (124.73 ± 1.9 mg QE/g) was also higher than FCL (91.5 ± 3.01 mg QE/g). The quantitative analysis of total flavonoids in JSL methanolic and ethanolic extracts revealed higher total flavonoid content, 13.83 mg QE/g and 15.66 mg QE/g, respectively, compared to those seen in FCL extracts, 7.37 and 8.37 mg QE/g. Ethanolic extracts of both plants showed a higher extraction of phytochemicals than methanolic extracts. Öztürk et al. showed the total flavonoid content of *J. sabina* methanolic plant extracts was lower, at 8.83 mg QE/g^27^. Zengin et al. showed that, for *F. halophila*, the highest flavonoid content was obtained from the acetone extract (34.52 mg RE/g extract), followed by the methanolic extract (24.13 mg RE/g extract), and then the chloroform extract (8.61 mg RE/g extract)^28^.

### Chemical content analysis by GC–MS

Using gas chromatography/mass spectrometry (GC–MS) analysis for the JSL ethanolic extract, the most abundant components were Mome inositol (48.71%), (-)-germacrene D (13.27%), (+)-totarol (7.68%), and cedrol (4.99%), while in the methanolic extract, mome inositol (91.43%), (+)-totarol (2.59%), and alpha-terpinyl acetate (2.40%) were seen. Sesquiterpenes were also the predominant constituents in both methanolic and ethanolic extracts (Table 2). According to GC–MS analysis of FCL ethanolic extract, the most abundant components were phytol (35.63%), trichloroacetic acid, tetradecyl ester (30.96%), dichloroacetic acid, tridecyl ester (27.66%) and gazaniolide (24.09%). The most abundant components of the methanolic extract were 1, 3, 4, 5-tetrahydroxycyclohexanecarboxylic acid (47.62%), octadecanoic acid (11.28%), and cytidine (6.26%). According to these results, organic compounds (25%) were the most abundant constituents for FCL methanolic extract (Table 3). Only a few studies have collected such data for these species. In a similar study by to Lamnaouer et al. the dried and ground leaves of *F. communis* var. genuine collected in Morocco were extracted successively with petrol and CH, Cl. Their results detected three new daucane sesquiterpenes related to jaeschkeanadiol^29^. Another study by Rahali et al., using RP-HPLC, showed that the most abundant components in methanolic extracts from the flower, fruits and stems of *F. communis* collected from north Tunisia were resorcinol, ferulic acid, and syringic acid, together with coumarin^30^. Orhan et al. used HPLC to check the chemical profile of ethanolic extracts from leaves of *Juniperus* species, showing the most abundant phytochemical constituents were amentoflavone and umbelliferone. Amentoflavone, a major compound in *J. foetidissima* and *J. sabina* leaves, was determined. Umbelliferone was detected only in the leaves, while fruits lacked this coumarin family compound^31^. Another study showed the chemical composition of methanolic extracts from female and male leaves of *J. sabina*. Both females and males showed no alkaloid or saponin content. However, a small quantity of tannins was detected in female *J. sabina* leaves and a moderate quantity in male leaves, while both male and female *J. sabina* plants showed a high flavonoid content^32^.

**Table 2:** Chemical composition of *Juniperus sabina* Leaves ethanolic and methanolic extract and their bioactivities.

Peak#	R.Time	Components	Extract type (%) peak area		Biological activity
			JSL ethanolic extract	JSL methanolic extract	
1	3.408	Alpha-Pinene	0.29	–	Antimicrobial, apoptotic, antimetastatic, and antibiotic
2	4.173	Beta-Ocimene	0.15	–	Anti-inflammatory, anti-viral, and anti-fungal
3	5.106	(+ -)-Linalool	0.08	–	Antibacterial, antimicrobial, antioxidant
4	6.637	Linalyl anthranilate	0.05	–	Antimicrobial, antibacterial
5	7.067	Acetic acid	0.17	–	Antibacterial and antifungal
6	7.657	Alpha-Terpinyl acetate	2.70	2.40	Antibacterial, antioxidant
7	8.444	Beta-Caryophyllene	1.43	–	Antimicrobial
8	8.780	Alpha-Caryophyllene	1.10	0.72	Antibacterial
9	8.826	Beta-Cubebene	0.85	–	Antimicrobial
10	8.913	Alpha-Amorphene	1.11	–	Antimicrobial, antioxidant
11	9.001	Germacrene D	13.27	0.73	Antibacterial
12	9.117	Naphthalene,1,2,4a,5,6,8a- hexahydro-4,7-	1.34	–	Antioxidant
13	9.284	( +)-Delta-cadinene	1.94	–	Antibacterial
14	10.185	Cedrol	4.99	–	Antibacterial, fungicidal activity
15	11.568	Mome inositol	48.71	91.43	antitumor activity
16	15.137	( +)-Totarol	7.68	2.59	Antibacterial, antimycobacterial, antileishmanial, antimalarial
17	15.221	Ferruginol	2.68	1.12	Antiviral
18	16.450	Totarolone	0.34	–	Antibacterial, Antihyperglycemic
19	16.520	(+)-Agathadiol	2.38	–	Antioxidant, Antibacterial,
20	19.887	Vitamin E	0.42	–	Antioxidant

**Table 3:** Chemical composition of *Ferula communis* Leaves ethanolic and methanolic extract and their bioactivities.

Peak #	R.Time	Components	Extract type (%) peak area		Biological activity
			FCL ethanolic extract	FCL methanolic extract	
1	4.246	Triethyl borate	7.99	–	Antimicrobial
2	8.567	Dimethyl ester of tartronic acid	–	0.97	Antibacterial
3	9.182	Tetraethyl ester	11.08	–	antioxidant and antimicrobial
4	10.165	1,3,4,5-Tetrahydroxy-cyclohexa	21.12	–	anti-tumor, anti-neurodegenerative, anti-inflammatory
5	12.167	1-Deoxy-d-arabitol	–	1.06	Antioxidant, Antibacterial
6	12.278	Catechol	–	2.33	Antibacterial
7	12.340	2,2'-Dimethoxydiphenylamine	–	0.86	Antibacterial
8	13.702	Phytol	35.63		Antioxidant, Antibacterial, Anti-inflammatory
9	13.788	Resorcinol-p-benzoquinone	–	0.51	Antibacterial, antioxidant,
10	14.029	Linoleic acid ethyl ester	1.29	–	Antioxidant, anti-tyrosinase
11	14.073	Ethyl linolate	2.57	–	Antioxidant
12	14.768	2,2-Diethylacetamide	–	0.55	Apoptotic
13	14.967	Dichloroacetic acid	27.66	–	Tumour-promoting
14	16.800	Trans-spinacene	15.30	–	Antinociceptive, anti-inflammatory
15	16.862	2-Cyclobutene-1-carboxamide	–	0.73	Antimicrobial
16	16.946	2,1,3-Benzothiadiazole	–	1.66	Antifungal
17	19.288	Thiophene	–	1.99	Antimicrobial
18	19.315	2,3-Dihydroindole-4-ol-2-one dibromo-3,3-dimethyl-1h-pyrazole	–	1.55	Antioxidant
19	19.666	1,3,4,5Tetrahydroxycyclohexanecar boxylic acid	–	47.62	Antioxidant
20	20.382	Trichloroacetic acid, tetradecyl ester	30.96	–	Antioxidant
21	20.579	Cyclohexene	–	1.17	Antibacterial
22	25.250	Tridecyl ester	12.97	–	Antioxidant, Anticancer, Antimicrobial
23	34.163	Octadecanoic acid	–	11.28	Anti-inflammatory
24	34.592	1-Ethyl-2-[(R)-(2-iodoethoxy)(2-isopropylphenyl)methyl]benzene	–	0.74	Antibacterial
25	34.637	Methomyl	–	0.39	Anticoagulant
26	34.689	1,1,1,3,3,5,5,7,7-Nonamethyltetras siloxane	–	0.21	Antimicrobial
27	34.847	3-(3-Chloro-phenyl)-3-oxo-propionic acid methyl ester	–	1.32	Antimicrobial

### Antioxidant activity

**Figure 1:**
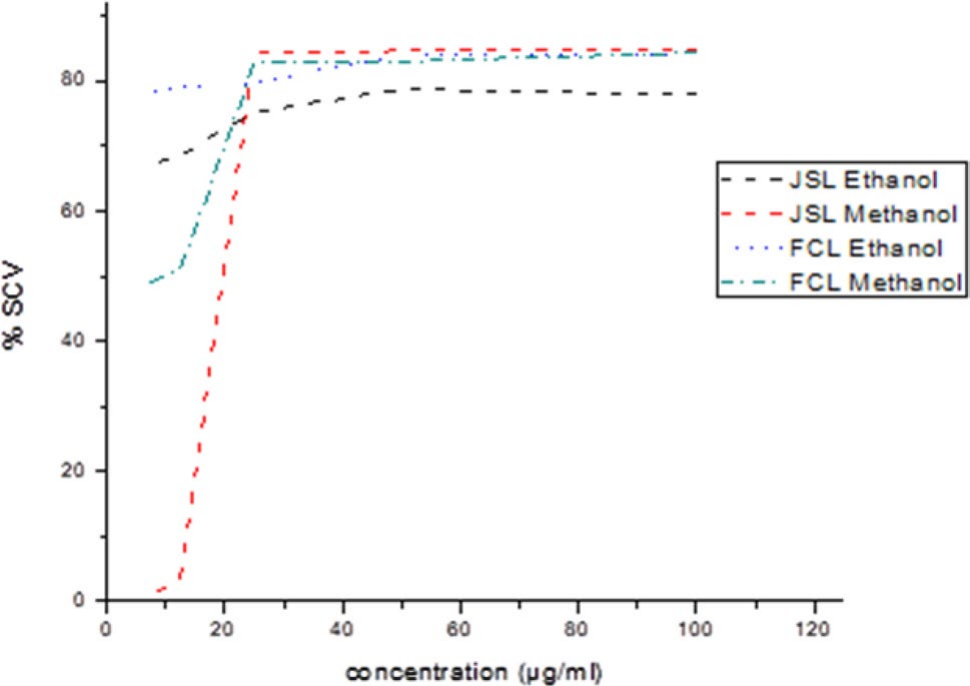
Comparison of percentage scavenging activity of Methanolic and Ethanolic extracts of *Juniperus sabina* and *Ferula communis* leaves using DPPH assay. (% SCV): Percentage of scavenging activity; (JSL) Juniperus sabina leaves; (FCL) Ferula communis Leaves.

The antioxidant activity was estimated using the 1, 1-diphenyl-2-picrylhydrazyl (DPPH) assay for different concentrations of methanolic and ethanolic extracts from both JSL and FCL. Both plants' extracts showed robust antioxidant activity compared with the ascorbic acid positive control. In this study, extracts from both plants showed high reducing power, with antioxidant activity increasing with the extract concentration. The percentage of scavenging activity of JSL methanolic extracts was determined at various concentrations: 6.25 (0.49 ± 0.02%), 12.5 (3.4 ± 0.02%), 25 (84.4 ± 0.06%), 50 (84.7 ± 0.01%), and 100 µg/ml (85 ± 0.01%). Scavenging activity was also determined for the same concentrations of methanolic extracts from FCL: 6.25 (48.26 ± 0.05%), 12.5 (51.09 ± 0.7%), 25 (82.87 ± 0.01%), 50 (83.03 ± 0.02%), and 100 µg/ml (84.4 ± 0.02%) (Fig. 1). Similarly, the percentage of scavenging activity for specific ethanolic extract concentrations from JSL was: 6.25 (67.3 ± 0.7%), 12.5 (68.5 ± 0.7%), 25 (75.2 ± 0.02%), 50 (78.8 ± 0.01%), and 100 µg/ml (77.9 ± 0.01%). Measurements were also determined for FCL ethanolic extracts: 6.25 (78 ± 0.7%), 12.5 (79.08 ± 0.02%), 25 (79.8 ± 0.1%), 50 (84.0 ± 0.01%), and 100 µg/ml (84.01 ± 0.5%). The DPPH assay percentage of inhibition with JSL extracts varied from 0.49 to 85%, while that of FCL extracts was 48.26–84.4%, for these 5 concentrations.

In a previous study, the DPPH assay percentage of inhibition for the methanolic extract of *J. Sabina* was 24.66%, 34.94%, and 55.49% for concentrations 25, 50, and 100 µg/ml respectively^27^. At the same concentrations, our research showed JSL had a higher percentage of inhibition at 84.4%, 84.7% and 85%, respectively. In another study, Jordanian *F. Communis* showed 86.7 µmol TE g^−1^ DW antioxidant activity^26^. Zengin et al. methanolic extracts from *F. halophila* also showed a high antioxidant activity of 95.28 ± 3.80% due to the polarity of methanol, enabling the extract to scavenge the DPPH radicals more actively^28^. Results from Rahali et al. revealed that the different organs of the *F. communis* plant had various antioxidant activities. Thus, flower extracts showed the highest DPPH-scavenging compared to stems and fruits, indicating the highest antioxidant capability was in this organ. This might be because it also contains the highest total phenolic content^30^. Natural plant antioxidants, including phenolic compounds, can prove beneficial by removing harmful free radicals. Therefore, phenolic compounds help to protect cells from oxidative damage. Chanjirakul et al. reported that plant extracts rich in phenolic compounds also provide antibacterial activity^33^. Results from another study confirm this result. According to Onyebuchi et al., extraction temperature significantly affected the total phenolic content and radical-scavenging properties of the different extracts^22^.

### Microbiological inhibition analysis

#### MIC antibacterial activity

In this study, the antimicrobial activity of methanolic and ethanolic extracts of JSL and FCL growing on the island of Cyprus was tested against two strains of gram negative bacteria, *Escherichia coli* O157:H7 (932) and *Salmonella typhimurium* (B-4420), and gram positive bacteria *Bacillus cereus* (ATCC 7064) and *Staphylococcus aureus* (6538 P). Both methanolic and ethanolic extracts of JSL and FCL showed effective antimicrobial results in minimal inhibitory concentration (MIC) assays. As shown in Table 4, the best MIC values were detected against *S. typhimurium* with all extracts, except for the FCL ethanolic extract. FCL methanolic extract had the best MIC against *E. coli*. The weakest antibacterial effect of all extracts was detected against *S. aureus*. The negative control containing nutrient broth did not show bacterial growth, while the positive control did.

**Table 4:** Antibacterial activity (MIC) of *Juniperus sabina* and Ferula communis on gram negative and gram positive bacteria using different extract concentrations.

Microorganisms	*E.coli* O157:H7 (932)					*S.typhimurium* (B-4420)					*B.cereus* (ATCC 7064)					*S.aureus* (6538 P)				
Concentration (mg/ml)	100	50	25	12.5	6.25	100	50	25	12.5	6.25	100	50	25	12.5	6.25	100	50	25	12.5	6.25
JSL EE	−	+	+	+	+	−	−	−	−	+	−	−	−	−	+	−	+	+	+	+
JSL ME	−	−	+	+	+	−	−	−	−	+	−	−	−	−	+	−	−	+	+	+
FCL EE	−	−	+	+	+	−	+	+	+	+	−	−	+	+	+	−	+	+	+	+
FCL ME	−	−	−	−	+	−	−	−	−	+	−	−	+	+	+	−	+	+	+	+
NB	−					−					−					−				
NNB	+					+					+					+				

Values are expressed as the mean ± SD (n = 3).

*EE* ethanol extract, *ME* methanol extract; Negative Control = NB: Nutrient Broth; Positive Control = NNB: Nutrient Broth with Bacteria; (+): Shows growth of Bacteria; (−): Shows NO growth of Bacteria.

Al-Yahya et al. showed petroleum ether crude extract from FCL had significant MIC antibacterial activity against *S. aureus*, *Bacillus subtilis*, *Streptococcus durans* and *Enterococcus faecalis* (MIC values 2.5, 2.5, 1.25 and 1.25, respectively)^34^. Additionally, Gamal et al. showed that ethyl acetate and n-butanol FCL extracts had considerable antimicrobial activity against *E. coli*, *Pseudomonas aeruginosa*, *B. cereus*, *S. aureus*, and *E. faecalis*^35^. MIC results of the n-butanol extract (MIC 2.0–4.0 mg/ml) revealed significantly greater antimicrobial activity compared to the ethyl acetate extract (MIC 8.0–12.0 mg/ml). However, according to Taviano et al., methanolic extracts from *Juniperus* spp. showed significant antimicrobial activity only against Gram-positive bacteria. No important antimicrobial activity was seen against Gram-negative bacteria or yeast. The most sensitive species to methanolic extracts of *Juniperus drupacea* Labil (MIC 9, 76) and *Juniperus oxycedrus* L. subsp. macrocarpa (MIC 4.88) was *S. aureus* followed by *Staphylococcus epidermidis* and *Enterococcus hirae*. The least sensitive species was *B. subtilis*^36^.

#### Disk diffusion method for antibacterial activity

The FCL ethanol extract (100 mg/ml) had the greatest antibacterial zone of inhibition (ZI) (16.5 mm) against *E. coli*, and the JSL ethanol extract (100 mg/ml) had the greatest ZI (12 mm) against *S. aureus*. Ethanolic extracts of both JSL and FCL were ineffective against *B. cereus*, with no activity seen. Chloramphenicol (10 μg/disc) and Amoxicillin (10 μg/disc) were used as positive controls to determine the sensitivity of microbial strains. Disks containing DMSO were used as a negative control. The highest ZI for methanolic extracts was from JSL (16.5 mm) against *S. aureus*, followed by the FCL methanolic extract against *E. coli* (12 mm), both at a concentration 100 mg/ml. FCL methanolic extract against *S. typhimurium* (Table 5) detected no antimicrobial activity.

**Table 5:** Antibacterial activity of different extract concentrations of *Juniperus sabina* and *Ferula communis* using disc diffusion method against gram negative and gram positive bacteria.

Microorganisms	*E.coli* O157:H7 (932)					*S.typhimurium* (B-4420)					*B.cereus* (ATCC 7064)					*S.aureus* (6538 P)				
Concentration (mg/ml)	100	50	25	12.5	6.25	100	50	25	12.5	6.25	100	50	25	12.5	6.25	100	50	25	12.5	6.25
JSL Ethanol Extract	9 ± 0	7.5 ± 0.7	7 ± 0	0	0	9.5 ± 0.7	7 ± 0	7 ± 0	0	0	0	0	0	0	0	12 ± 0	11 ± 0	0	0	0
JSL Methanol Extract	8.5 ± 0.7	7 ± 0	0	0	0	10.5 ± 0.7	7 ± 0	0	0	0	10 ± 0	9 ± 0	0	0	0	16.5 ± 0.7	7 ± 0	0	0	0
FCL Ethanol Extract	16.5 ± 2.1	9.5 ± 0.7	7 ± 0	0	0	9.5 ± 0.7	0	0	0	0	0	0	0	0	0	9.5 ± 0.7	0	0	0	0
FCL Methanol Extract	12 ± 1.4	8.5 ± 0.7	0	0	0	0	0	0	0	0	7 ± 0	0	0	0	0	9 ± 0	0	0	0	0
Amoxicillin	17.5 ± 0.7					17 mm ± 0					21 ± 0					20 mm ± 0				
Chloramphenicol	22 mm ± 0					22 mm ± 0					20 mm ± 0					27 mm ± 0				
Disc With DMSO only	0 mm					0 mm					0 mm					0 mm				

(0) non-activity; 7 < IZ < 9.9 mm: Slight activity; 10 < IZ < 11.9 mm: Moderate activity; 12 < IZ < 15 mm: High activity; 15 mm < IZ: Strong activity. Values are expressed as the mean ± SD (n = 3).

Öztürk et al. showed the antibacterial effect of the *J. sabina* methanolic extract against several bacterial strains, with no ZI detected against *E. coli*, and an 8.5 mm ZI detected against *S. aureus*^27^. The best results for inhibiting bacterial growth were seen on multiple-antibiotic-resistant *Staphylococci* and some strains of multiple-antibiotic-resistant *Stenotrophomonas maltophilia*. In this research, JSL ethanolic and methanolic extracts had an effective antibacterial activity against *E. coli* (ZI = 8.5 mm) and *S. aureus* (ZI = 16.5 mm), respectively. In another study, the methanolic extract of *J. phoenicea* was effective for inhibiting the growth of *E. coli* and *S. aureus*, with ZI size increasing with extract concentration; as the concentration increased from 20, 30, to 40%, the ZI measured 11, 12 and 13 mm for *E. Coli* and 15, 17 and 20 mm for *S. aureus*, respectively^23^. Sitotaw et al. showed that the in vivo antibacterial activity of root and stem methanolic and ethanolic extracts of *F. communis* varied by concentration (100 mg/ml and 200 mg/ml) and that the methanolic extract showed higher antibacterial activity against *S. aureus* and *E. coli* than ethanolic extracts. Significantly higher antibacterial activity was detected using 200 mg/ml of extracts. However, they also showed that *F. communis* ethanolic stem extracts could inhibit the growth of *E. coli* at 100 mg/ml concentration with a minimal ZI (8.00 ± 0.00 mm)^37^. These findings show that extracts from different parts of the plant have other antibacterial activity. This might be due to the distribution of active ingredients between various plant parts, whether in leaves, stems or roots.

### In vitro cytotoxic effects of JSL and FCL methanolic and ethanolic extracts on MDA-MB-231 and MCF-7 breast cancer cell lines

Plant polyphenols protect cells from apoptosis^38^ and thus have anticancer properties by inhibiting cell proliferation^39,40^ which brings about various biological effects. To test the anticancer properties of the methanolic and ethanolic extracts from JSL and FCL, we applied them in multiple concentrations to two breast cancer cell lines.

**Figure 2:**
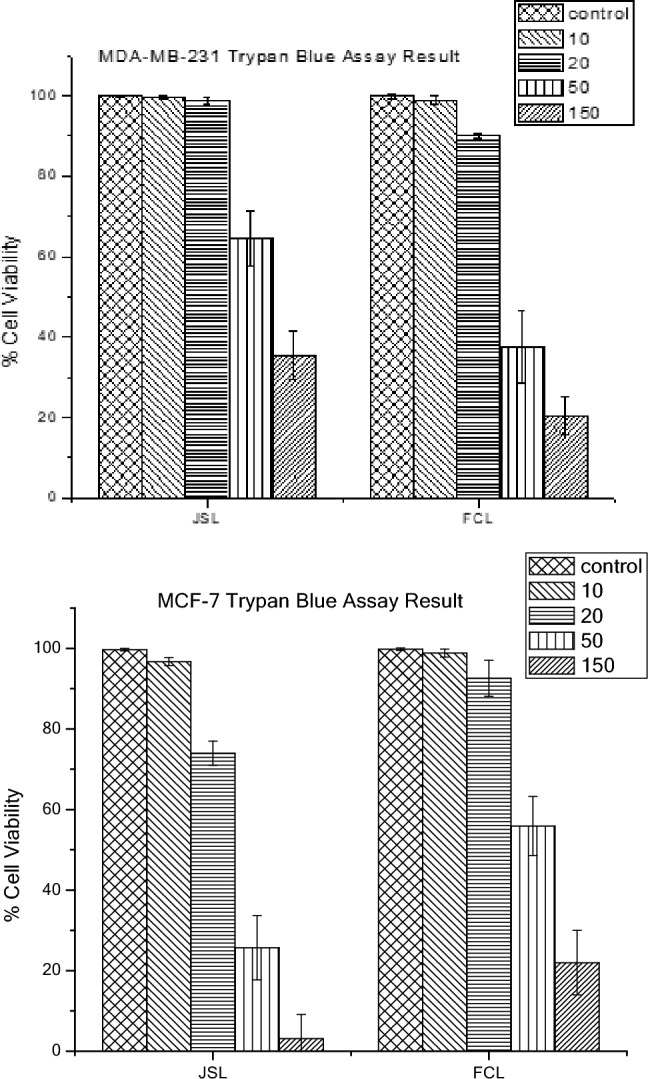
JSL and FCL methanolic extracts cause a significant anti-proliferative effect on MCF-7 and MDA-MB-231 cells. Increasing Concentration-effect of JSL and FCL methanolic extract treatment 10–150 μg/ml for 24 h in MCF-7 and MDA-MB-231 cells. Data are represented as mean ± S.D. Statistical significance: p < 0.05 vs control MCF-7 vs MDA-MB-231 according to Student’s t-test.

The application of increasing concentrations of JSL and FCL methanolic extracts (10, 20, 50 and 150 µg/ml vs. control) showed a significant decrease (p < 0.05, n = 9) in the cellular viability of both breast cancer lines dependent on concentration. This may be due to increased bioactive compounds in the extract as the concentration increases. JSL methanolic extract treatment on MDA-MB-231 (10 µg/ml: 99.47%; 20 µg/ml: 98.62%; 50 µg/ml: 64.5%; 150 µg/ml: 35.5%) and MCF-7 (10 µg/ml: 96.7%; 20 µg/ml:74%; 50 µg/ml: 25.7%; 150 µg/ml: 3.1%) caused a significant reduction in cell viability, showing the JSL methanolic extracts had increasing cytotoxic effect on the selected cancer cell lines as the concentration increased. FCL methanolic extract treatment had a similar impact on MDA-MB-231 (10 µg/ml: 98.78%; 20 µg/ml: 90.01%; 50 µg/ml: 37.45%; 150 µg/ml: 20.4%) and MCF-7 (10 µg/ml: 98.81%; 20 µg/ml: 92.56%; 50 µg/ml: 55.9%; 150 µg/ml: 22%) (Fig. 2).

**Figure 3:**
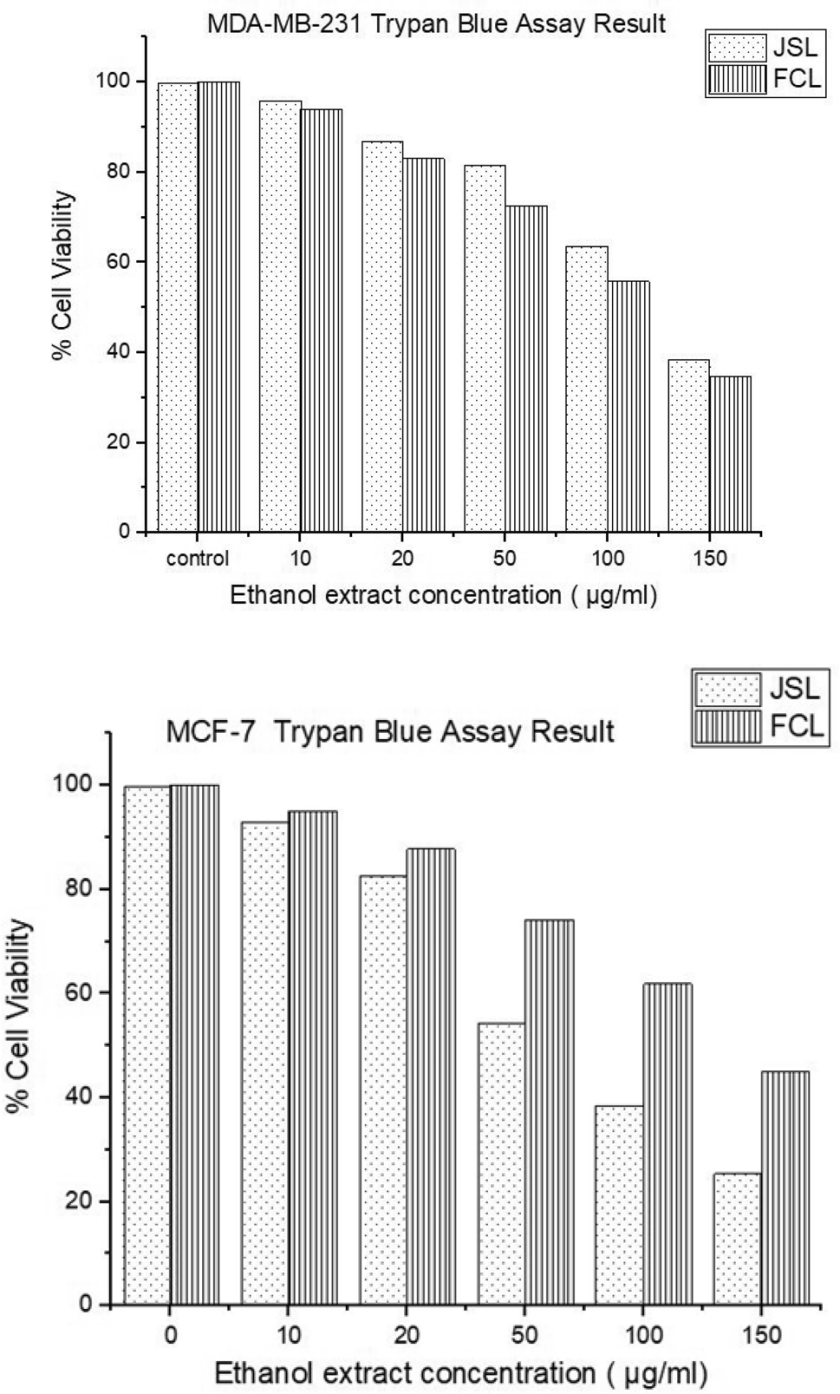
JSL and FCL ethanolic extracts cause significant anti-proliferative effects on MDA-MB-231 cells and MCF-7. Increasing Concentration-effect of JSL and FCL ethanolic extract treatment 10–150 μg/ml for 24 h in MCF-7 and MDA-MB-231 cells. Data are represented as mean ± S.D. Statistical significance: p < 0.05 vs control MCF-7 vs MDA-MB-231 according to Student’s t-test.

Similar results were seen for ethanolic extracts, where an increase in concentration (10, 20, 50 and 150 µg/ml vs control) showed a significant decrease (p < 0.05, n = 9) in cellular viability. JSL ethanolic extract treatment was effective on MDA-MB-231 (10 µg/ml: 96%; 20 µg/ml: 87%; 50 µg/ml: 81.5%; 150 µg/ml: 38.6%) and MCF-7 (10 µg/ml: 93%; 20 µg/ml: 82.8%; 50 µg/ml: 54.3%; 150 µg/ml: 25.4%). FCL ethanolic extract treatment had a similar effect on MDA-MB-231 (10 µg/ml: 94%; 20 µg/ml: 83%; 50 µg/ml: 72.6%; 150 µg/ml: 34.7%) and MCF-7 (10 µg/ml: 95%; 20 µg/ml: 87.8%; 50 µg/ml: 74.2%; 150 µg/ml: 44.9%) (Fig. 3).

**Figure 4:**
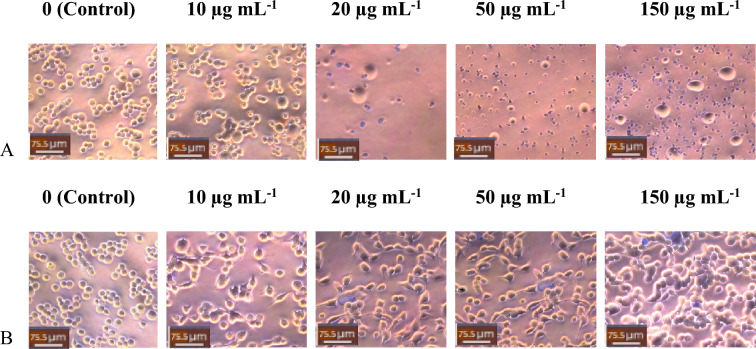
MDA-MB-23 cells Light microscope images (20x) of treatment with varying concentrations of samples: (**A**) JSL methanolic extract, (**B**) FCL methanolic extract. Control group = DMEM (left panel). Scale bars = 75.5 μm.

**Figure 5:**
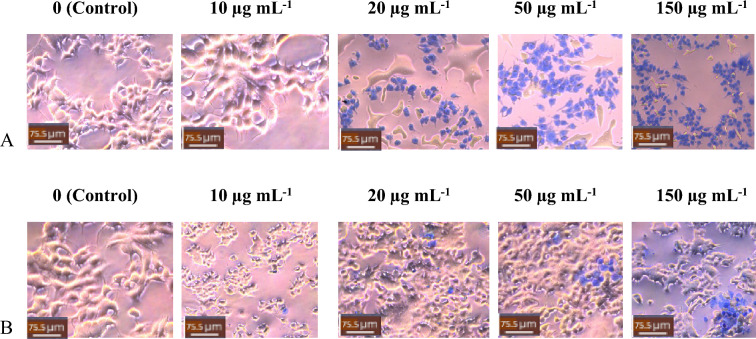
MCF-7 cells Light microscope images (20x) of treatment with varying concentrations of samples: (**A**) JSL methanolic extract, (**B**) FCL methanolic extract. Control group = DMEM (left panel). Scale bars = 75.5 μm.

Methanolic extracts from FCL showed a more significant reduction in cellular viability on MDA-MB-231 than that of JSL, as shown in Fig. 4. Contrastingly, the JSL methanolic extract caused the highest reduction of cellular viability for MCF-7, shown in Fig. 5.

**Figure 6:**
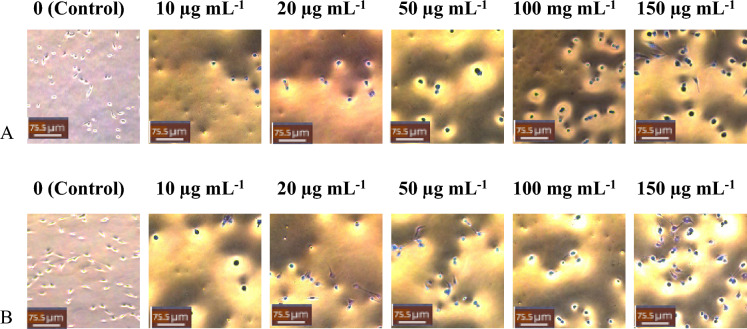
MDA-MB-23 cells Light microscope images (20x) of treatment with varying concentrations of samples: (**A**) JSL ethanolic extract, (**B**) FCL ethanolic extract. Control group = DMEM (left panel). Scale bars = 75.5 μm.

**Figure 7:**
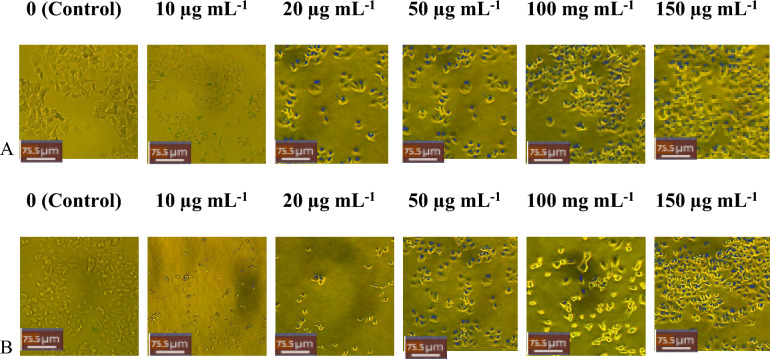
Images of MCF-7 cells treated with (**A**) JSL ethanolic extract, (**B**) FCL ethanolic extract. Scale bars = 75.5 μm. Control group = DMEM (left panel).

FCL ethanolic extract showed a lower cellular viability percentage than JSL ethanolic extract against MDA-MB-231 (Fig. 6). However, for ethanolic extracts, JSL showed better cytotoxic results overall at higher concentrations (Fig. 7). The most significant reduction was observed for all experiments with the 150 µg/ml concentration of the JSL methanolic and ethanolic extracts applied to MCF-7.

Our findings showed methanolic and ethanolic extracts from JSL and FCL had significant antiproliferative activity against breast cancer cell lines MDA-MB-231 and MCF-7. No other data has been recorded against these specific cell lines with extracts from our selected plants. Another study showed cytotoxic activities against Hela and MDA-MB-468 cells from fruit extracts and branchlets of male and female Iranian *J. sabina*^32^. Also, total extracts from the aerial parts of *J. sabina* had promising hepatoprotective activity against CCl_4_-induced toxicity in rats^41^. In another study, the cytotoxicity of ferulenol, isolated from *F. communis,* on human breast cancer (MCF-7), ovarian cancer (SKOV-3), leukemic cancer (HL-60), and colon cancer (Caco-2) cells was measured, showing cytotoxic effects at concentrations of 10 nM, 100 nM and 1 µM against these cancer cell lines^42^. One of the *Ferula* species, *F. szowitsiana* showed a solid cytotoxic property against MCF-7 cell line (IC50: 61.3 µg/ml)^43^. Additionally, root hexane extracts of *F. hermonis* showed a dose-dependent cytotoxic effect against MDA-MB-231 (IC50: 18.2 μg/ml)^44^.

Only a few studies have compared the cytotoxic effects of methanolic and ethanolic extracts for *J. Sabina*. In Sadeghi-Aliabadi et al., ethanolic extracts of *J. Sabina* branchlets were tested for cytotoxic effect against MDA-MB-468 cells using 3 different concentrations, 5, 10, and 20 μg/ml. Still, they did not show any cytotoxic activity^45^. One study on Juniper species *J. foetidissima* reported the cytotoxic effects of nardosinen extracted in the acetone extract from leaves and branchlets tested against various cancer cells, showing the cytotoxic effect against MCF-7 was dose-dependent^46^.

## Conclusions

A comparative study was conducted on methanolic and ethanolic extracts from *J. Sabina* and *F. communis* concerning phenolic profile, flavonoid profile, antioxidant activity, antimicrobial activity, and antiproliferative activity. For antioxidant activity JSL methanolic extracts at the tested concentrations showed a DHHP scavenging percentage of 0.49–85%, while that of FCL was 48.26–84.4%. Ethanolic extracts for JSL and FCL varied from 67.3–77.9% to 78–84.0%, respectively. The most dominant phytochemical in the ethanolic extract determined by GC–MS analysis of JSL was sesquiterpene, followed by diterpene alcohol, phenols, and esters. The highest zone of inhibition, 16.5 mm, was obtained with the FCL ethanol extract against *E. coli*, while no results were obtained against *B. cereus*. The FCL methanol extract showed the highest anticancer activity against MDA-MB-231 (20.4% viability) while the JSL methanolic extract was most effective on MCF-7 (3.1%). With the ethanolic extract, we determined higher antiproliferative activity for the FCL extract on MDA-MB-231 (34.7%), and the JSL extract on MCF-7 (25. 4%). The results of this study show that these endemic plant species can be evaluated as a source of natural compounds with cytotoxic and antimicrobial agents, which may be suitable for medical applications.

## Data Availability

All data is included in the submitted manuscript file.

## Abbreviations

JSL: *Juniperus **sabina* leaves
FCL: *Ferula** communis* leaves
CO2: Carbon dioxide
DMEM: Dulbecco’s modified eagle medium
FBS: Fetal bovine serum
GC–MS: Gas chromatography mass spectroscopy
WHO: World Health Organization
DPPH: 2,2-Diphenyl-1-picrylhydrazyl
MIC: Minimum inhibitory concentration
DMSO: Dimethyl sulfoxide
GAE: Gallic acid equivalent
QE: Quercetin equivalent
ZI: Zone of inhibition
Hrs.: Hours
T°C: Temperature
%SVC: Percentage of scavenging activity
