# Author Correction: Investigation of composition, antioxidant, antimicrobial and cytotoxic characteristics from *Juniperus sabina* and *Ferula communis* extracts

**DOI:** 10.1038/s41598-024-77743-6

**Published:** 2024-11-08

**Authors:** Doga Kavaz, Razan El Khaled El Faraj

**Affiliations:** 1 Department of Bioengineering, Cyprus International University, Northern Cyprus Via Mersin 10, Haspolat, Nicosia Turkey; 2 Biotechnology Research Centre, Cyprus International University, Northern Cyprus Via Mersin 10, Haspolat, Nicosia Turkey

Correction to: *Scientific Reports* 10.1038/s41598-023-34281-x, published online 03 May 2023

The original version of this Article contained an omission in the name of Razan El Khaled El Faraj, which was incorrectly given as Razan El Faraj.

The original Article has been corrected.

